# Tumour Single‐Cell Bioinformatics: From Immune Profiling to Molecular Dynamics

**DOI:** 10.1111/jcmm.70783

**Published:** 2025-09-15

**Authors:** Jane Siu‐fan Li, Zoey Zeyuan Ji, Aaron Qi Zhang, Calvin Sze‐Hang Ng, Guibin Qiao, Dongmei Zhang, Chunjie Li, Qing Zhang, Ka‐Fai To, Patrick Ming‐Kuen Tang

**Affiliations:** ^1^ Department of Anatomical and Cellular Pathology, State Key Laboratory of Translational Oncology The Chinese University of Hong Kong Hong Kong Hong Kong; ^2^ Department of Surgery The Chinese University of Hong Kong Hong Kong Hong Kong; ^3^ Department of Thoracic Surgery, Zhujiang Hospital Southern Medical University Guangzhou China; ^4^ College of Pharmacy Jinan University Guangzhou China; ^5^ Department of Head and Neck Oncology, West China Hospital of Stomatology Sichuan University Chengdu China; ^6^ Cancer Institute Xuzhou Medical University Xuzhou China; ^7^ Li Ka Shing Institute of Health Sciences The Chinese University of Hong Kong Hong Kong Hong Kong; ^8^ Peter Hung Pain Research Institute The Chinese University of Hong Kong Hong Kong Hong Kong

**Keywords:** immune landscape, single‐cell RNA sequencing, tumour microenvironment

## Abstract

Single‐cell RNA sequencing (scRNA‐seq) has transformed our understanding of tumours by enabling high‐resolution profiling of their cellular composition. Traditionally perceived as masses of homogeneous cancer cells, tumours are now recognised as complex ecosystems shaped by the tumour microenvironment (TME), which includes diverse immune cells, cancer‐associated fibroblasts and extracellular matrix components. scRNA‐seq has revealed remarkable heterogeneity within the TME, identifying novel or rare immune cell subsets and delineating their dynamic functional states. In particular, it has illuminated intercellular signalling networks and temporal cell‐state transitions that drive tumour progression and immune evasion. Moreover, the integration of scRNA‐seq data with clinical information has highlighted its potential in improving patient stratification, biomarker discovery and therapeutic target identification. Here, we systematically summarise recent advances in applying scRNA‐seq to dissect the TME, discuss the implications of these findings for immunotherapy resistance and precision oncology, and outline future opportunities for integrating scRNA‐seq with emerging technologies to develop more effective and personalised cancer treatment strategies.

## 1 Introduction

Cancer remains one of the leading causes of global mortality, presenting a persistent challenge to improve human life expectancy [1]. Despite the medical advances and breakthroughs made in recent years, the global cancer burden is still growing and is anticipated to grow over the next 50 years [2, 3]. Over the past few decades, our understanding of cancer has undergone a significant transformation. Once viewed as a mass of relatively homogenous cancer cells, tumours are now recognised as complex ecosystems known as the TME [4]. This intricate milieu comprises multiple components, including tumour‐infiltrating immune cells, cancer‐associated fibroblasts (CAFs) and extracellular matrix [4, 5].

In recent years, tremendous research efforts have been made to delineate the role of the TME in cancer progression [6]. Increasing evidence has shown that the TME facilitates cancer invasion and metastasis through various mechanisms, such as angiogenesis, extracellular matrix remodelling and immunosuppression [7, 8], which are now considered hallmarks of cancer [4]. A major challenge in studying the TME is tumour heterogeneity, encompassing both intratumoral and intertumoral heterogeneity [5, 9]. Tumour heterogeneity is observed not only at the phenotypic level but also at the genetic level, further underscoring the complexity of dissecting the TME [10, 11]. Among the various components of the TME, immune cells play a particularly important role, exhibiting both pro‐tumour and anti‐tumour effects. The infiltration of different immune cell populations has been associated with cancer progression, suggesting that tumour‐infiltrating immune cells could serve as valuable prognostic biomarkers and therapeutic targets [12, 13, 14, 15]. Consequently, the immune landscape of the TME has emerged as a critical focus of research.

Traditional biological techniques, which provide an overall view of bulk tumour samples, are inadequate for analysing specific cell types within the tumour. However, advances in next‐generation sequencing have revolutionised our approach to interpreting complex TME. scRNA‐seq has emerged as a state‐of‐the‐art technique enabling researchers to study the TME at single‐cell resolution [16]. It is a powerful genomic technique that allows researchers to analyse gene expression profiles of individual cells [17], facilitating the generation of detailed cell atlases of the TME. Since each cell is unique in both time and space, scRNA‐seq provides crucial insights into cellular heterogeneity [18].

**FIGURE 1:**
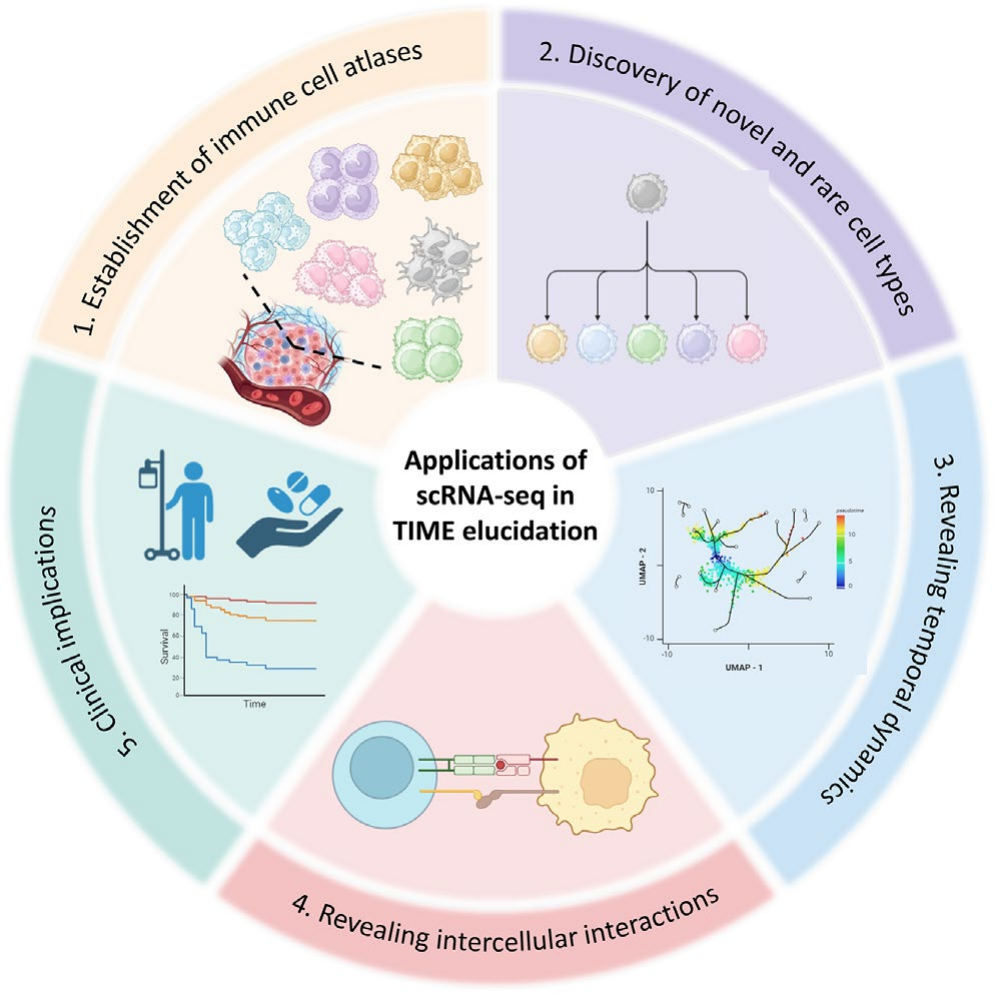
The Applications of scRNA‐seq in dissecting the TME. scRNA‐seq serves as a powerful tool for elucidating the complexity of the TME by enabling: (1) the establishment of comprehensive immune cell atlases, mapping the diverse immune landscape within tumours; (2) the discovery of novel and rare immune cell populations with potential functional significance; (3) the characterisation of temporal dynamics, capturing cellular transitions and lineage differentiation over time; (4) the identification of intercellular interactions, uncovering key signalling networks that shape the immune response; and (5) clinical applications, including biomarker discovery, patient stratification and the development of targeted immunotherapies.

These can be achieved through inference of cell–cell interactions and cellular trajectories in tumours with the aid of constantly emerging analytical tools for scRNA‐seq. In addition, the information obtained from scRNA‐seq studies of TME has valuable clinical implications, including the development of novel therapies, identification of biomarkers and improved patient stratification. This review aims to explore the diverse applications of scRNA‐seq in unravelling the internal architecture of the TME (Figure 1), drawing upon relevant studies to illustrate its potential in advancing our understanding of the TME.

## 2 Establishment of Immune Cell Atlases in TME

TME is a complex and heterogeneous ecosystem comprising various immune cell types that play pivotal roles in cancer progression. Several studies have utilised scRNA‐seq to dissect the TME, providing detailed views of the cellular landscape within tumours, including the immune components, establishing comprehensive immune cell atlases.

One remarkable finding from the establishment of immune cell atlases is the distinct compositions of immune cell populations observed across different cancer types. For instance, an early study by Chung et al. conducted scRNA‐seq of 515 cells from 11 breast cancer patients, identifying three main groups of immune cells, which were B cells, T cells and macrophages [19]. Another important landmark study of breast cancer revealing a diverse array of immune cell subpopulations by Azizi et al. produced a more comprehensive immune cell atlas with 45,000 immune cells from eight breast carcinoma patients [20]. Notably, the study revealed the heterogeneity as well as continuum of activation and differentiation states for T cells.

A comprehensive immune cell map of another cancer type, lung cancer, was also established in 2018 by Lambrechts et al. via scRNA‐seq of 52,698 cells from lung cancer patients [21]. The authors generated a comprehensive single‐cell map of the TME, including the immune components. Among different immune components, B cells were observed to be the most heavily enriched. Immune cell profiling also revealed different immune subpopulations and their features, such as low expression of CD8^+^ T cell marker genes linked with improved survival in lung adenocarcinoma (LUAD), but worse survival in lung squamous cell carcinoma (LUSC). A more recent and comprehensive scRNA‐seq study of 42 advanced NSCLC biopsy samples characterised various components of the TME and their interactions through intercellular signalling networks [22]. Some rare immune cell subtypes in tumours were also identified, such as follicular dendritic cells and T helper 17 (Th17) cells.

Immune cell atlas of melanoma generated by scRNA‐seq had already been available as early as 2016 by Tirosh et al. via scRNA‐seq of samples from 19 patients [23]. Detailed characterisation of various components of the TME uncovered their potential interactions through intercellular signalling networks. The analysis of tumour‐infiltrating T cells revealed exhaustion programmes, their connection to T cell activation and clonal expansion, and their variability across patients. The pivotal role of T cells in cancer progression and the effects of immunotherapies have been underscored in various cancer studies, emphasising the need for detailed T cell maps in addition to immune cell maps. For instance, Li et al. conducted a study that used T cells and NK cells isolated from samples of 25 melanoma patients to perform more in‐depth T cell analyses. Their results were consistent with previous scRNA‐seq studies, showing a wide differentiation spectrum from early dysfunction toward highly dysfunctional T cells, rather than discrete T cell populations [24]. Such widely observed phenomena suggest that this is a universal feature of T cells, rather than patient‐specific. The authors also observed that dysfunctional signatures were more prominent in CD8^+^ T cells from tumours than those in peripheral blood, indicating that such a dysfunctional state is induced locally within the TME.

In addition to the studies mentioned, numerous researchers have made significant efforts in characterising the TME of various cancer types, such as head and neck squamous cell carcinoma (HNSCC) [25], hepatocellular carcinoma (HCC) [26], colorectal cancer (CRC) [27], pancreatic ductal adenocarcinoma (PDAC) [28] and osteosarcoma [29]. All these studies utilised scRNA‐seq to dissect the TME, identifying distinct subpopulations or functional states of immune cells by revealing the unique immune landscape of each cancer type. These contributions have significantly enhanced our understanding of tumour biology and have important therapeutic implications.

Based on the high‐resolution maps of the immune landscapes within the TME of different cancer types, detailed atlases have served as valuable reference points for scRNA‐seq pan‐cancer analyses, enabling the identification of shared and unique features across different cancers. These pan‐cancer analyses differed in their specific focus areas, such as immune cell subsets, cancer cell classification and the whole cellular ecosystem. For example, Gavish et al. integrated data from 77 scRNA‐seq studies, analysing 1163 tumour samples across 24 cancer types to provide a broad view of transcriptional intratumour heterogeneity (ITH). Although this study focused on examining ITH of malignant cells, it provided a large‐scale immune cell map, showing heavy infiltration of macrophages, T cells and B cells compared to other immune components [23]. They also studied the cytotoxicity and recruitment of T cells; for instance, they observed a strong correlation between CD8^+^ T cell cytotoxicity and macrophage interferon response. A more recent large‐scale analysis of scRNA‐seq datasets from over 1000 tumours across 30 cancer types, including matched normal tissues, was performed by Kang et al. [24]. This study had greater emphasis on the TME; a major finding is the identification of gene signatures and immune components of tertiary lymphoid structures, which are ectopic aggregations of lymphocytes and had previously been correlated with favourable immunotherapy responses [25]. They also identified several cell states that are linked to poor responses to immunotherapy; for example, CTSK^+^ macrophages [24]. By revealing shared and cancer‐specific features, or treated and untreated features, large‐scale pan‐cancer analyses have provided valuable information for future development in precision oncology.

Apart from the pan‐cancer analyses mentioned, the importance of the TME has been further emphasised and shown by efforts made in conducting scRNA‐seq pan‐cancer analyses of specific immune cell types, such as NK cells [26], myeloid cells [27], and T cells [28]. Tang et al. integrated scRNA‐seq data from over 700 patients across 24 cancer types, generating a comprehensive tumour‐infiltrating NK cell atlas. The authors examined NK cell heterogeneity in a cancer type‐specific manner; for example, immature CD56^bright^CD16^lo^NK cells were observed to be enriched in nasopharyngeal cancer (NPC) and basal cell carcinoma [26].

In addition, some universal features were reported, such as reduced CD56^dim^CD16^hi^ NK cell level in most cancer types, and dysfunctional states with impaired cytotoxicity, named TaNK cells. Another more recent pan‐cancer NK cell study also identified CD56^bright^ and CD56^dim^ NK cells as the major subtypes in the TME, with CD56^bright^ NK cells being less mature and CD56^dim^ NK cells being more cytotoxic [29]. These subsets were found to represent different states along a differentiation continuum, transitioning from CD56^bright^ to CD56^dim^ NK cells. Further subclassification revealed several functionally distinct subtypes, such as DNAJB1^+^CD56^dim^CD16^hi^ NK cell, stressed CD56^bright^ NK cells, effector CD56^dim^ NK cells and adaptive CD56^dim^ NK cells [26, 29].

Pan‐cancer studies of other immune cell types, such as neutrophils, have also revealed their heterogeneity. Wu et al. integrated scRNA‐seq data from 143 patients across 17 cancer types to profile neutrophils and identified 10 distinct states with specialised functions, including angiogenesis, chemotaxis and antigen presentation [30]. Notably, one key finding was the identification of HLA‐DR^+^CD74^+^ neutrophils as potential immunotherapy via antigen presentation to stimulate T cells, offering an alternative antigen‐presenting strategy in the immunosuppressive TME where classical APC cells, such as DCs with HLA downregulation, are dysfunctional. The phenotypic and functional heterogeneity of neutrophils was also examined in another study that integrated human and murine scRNA‐seq datasets, while only a few cancer types were studied. Two main subtypes: an activating subtype resembling healthy neutrophils and a tumour‐specific subtype were identified [31]. This study also implicated the IL1β/CXCL8/CXCR2 signalling axis as a driver of neutrophil transition from healthy to tumour‐promoting and metastatic states.

B cells have likewise been the subject of extensive pan‐cancer analyses in 2024. Ma et al. analysed scRNA‐seq data of B cells obtained from 269 patients across 20 cancer types using scRNA‐seq, complemented with single‐cell assay for transposase‐accessible chromatin sequencing (scATAC‐seq) and single‐cell B cell receptor sequencing to generate a multi‐omic atlas of intratumoral B cells [32]. 15 B cell subsets were identified, following either an extrafollicular (EF) or germinal center (GC) developmental pathway. Four of these subsets were previously uncharacterised: interferon‐stimulated gene‐positive naïve B cells, stressed B cells with elevated expression of heat shock proteins and hypoxia‐related genes, pre‐GC B cells and atypical memory (AtM) B cells with high DUSP4 expression. Importantly, the balance between EF and GC B cell responses was linked to disease progression and clinical outcomes. For instance, AtM B cells arising from the EF pathway were associated with immunosuppressive activity and poor prognosis. Shortly afterward, another study analysed B cells from 649 patients across 19 cancer types [33]. This study also reported the presence of DUSP4^+^ AtM B cells observed in the study of Ma et al., while they reported the DUSP4^+^ AtM B cells as two subpopulations: FCRL4^+^ atypical B cells (ABCs) and PDCD^+^ ABCs. They emphasised the importance of the identification of stress‐response memory B cells and ABCs, especially FCRL4^+^ ABCs, which were characterised as highly proliferative with significant clonal expansion and were observed to interact with CXCL13^+^CD4^+^ T cells, offering a potential biomarker for predicting immunotherapy response. Fitzsimons et al. further corroborated and refined this pan‐cancer portrait by characterising phenotypically distinct B cell subtypes. Consistent with Ma et al., this study also reported the presence of AtM B cells with high DUSP4 expression, and a group of activated B cells that were observed in three activated B cell clusters by Ma et al. [34]. Together, these pan‐cancer B cell studies provide comprehensive atlases that serve as valuable references for understanding intratumoral B cell diversity and function.

Similarly, Cheng et al. used myeloid cells from 210 patients across 15 cancer types to create a large‐scale tumour‐infiltrating myeloid cell map, demonstrating the highly heterogeneous nature of tumour‐infiltrating myeloid cells and revealing their shared and cancer‐specific characteristics. For example, LAMP3^+^ conventional DCs (cDCs) were observed to be widely present in different types of cancer, and greater abundance of TNF^+^ mast cells was found in NPC [27]. They also observed diverse macrophage subsets across different cancer types, such as SPP1^+^ tumour‐associated macrophages (TAMs), C1QC^+^ TAMs, and FN1^+^ TAMs, which were enriched in different cancer types. Pan‐cancer analysis of another immune component, T cell, was performed by Zheng et al. using scRNA‐seq data of T cells from 316 patients with 21 cancer types, aiming to build a high‐resolution pan‐cancer T cell atlas for global inference of tumour‐associated T cells [28]. The authors identified various T cell subpopulations and functional states, as well as potential biomarkers or gene signatures associated with these populations or states; for instance, FAT1 mutations are positively correlated with TNFRSFP^+^ T regulatory cells (Tregs). The findings could suggest potential avenues for developing or improving T cell‐based immunotherapies.

**FIGURE 2:**
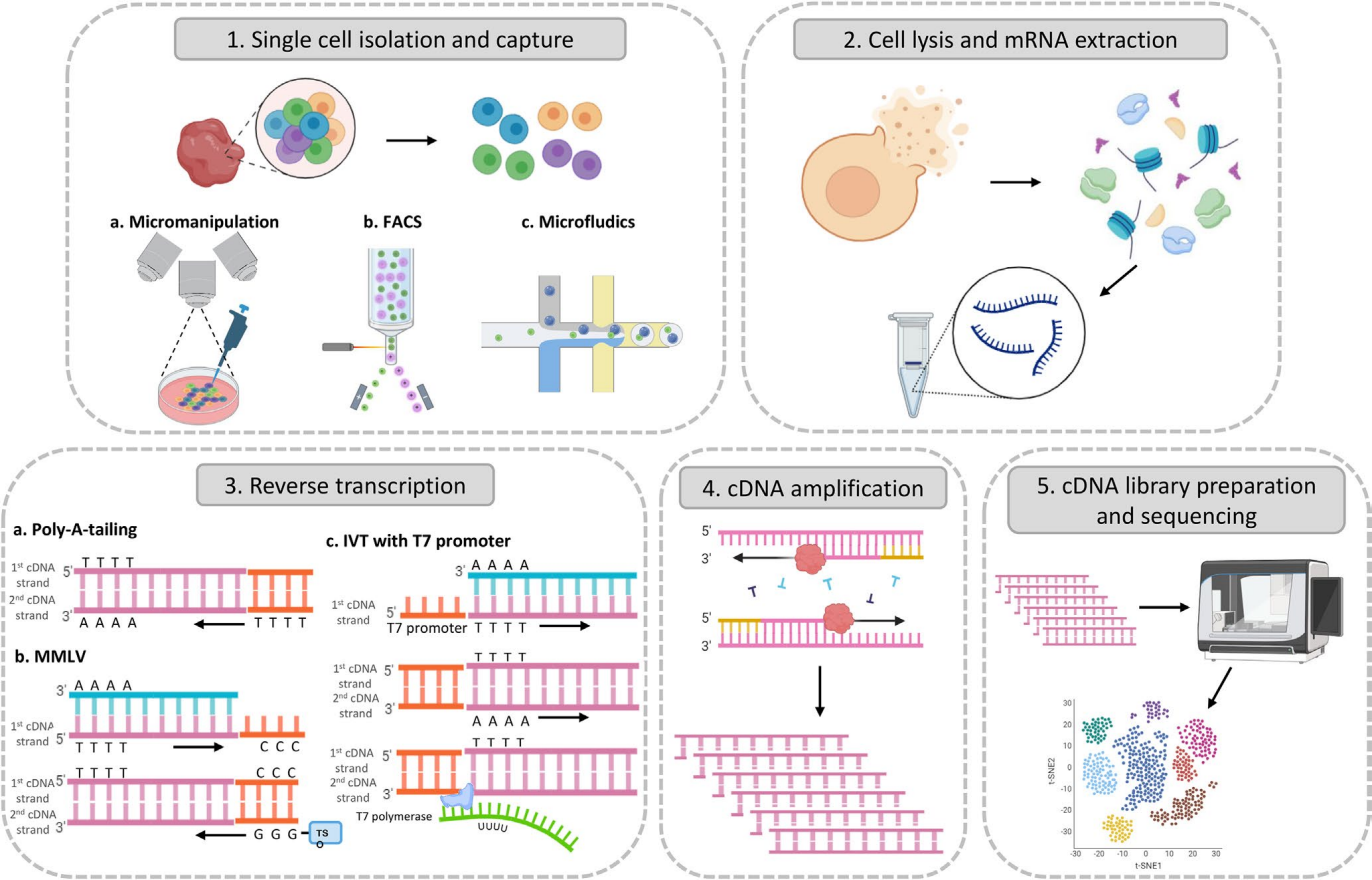
The general procedures of scRNA‐seq. The general procedures of scRNA‐seq include (1) single cell isolation and capture, (2) cell lysis and mRNA extraction, (3) reverse transcription, (4) cDNA amplification and (5) cDNA library preparation and sequencing.

All these scRNA‐seq studies have generated a wealth of complex data about the TME. A typical scRNA‐seq workflow is illustrated in Figure 2, outlining the key steps involved in the generation and analysis of single‐cell transcriptomic data. To handle this vast and intricate data, researchers have made significant efforts to curate and organise available scRNA‐seq datasets, generating public digital platforms that incorporate advanced tools for their analysis and visualisation [16, 35]. The mainstream protocols used in these studies, including their methodologies and technical approaches [36, 37, 38, 39, 40, 41, 42, 43], are summarised in Table 1. One such pioneering effort is the interactive web‐based tool developed by Cheng et al., providing access to the myeloid atlas generated from the pan‐cancer myeloid cell analysis [27]. Similarly, the Tumour Immune Single Cell Hub (TISCH) has emerged as a large‐scale platform encompassing 76 datasets from 27 cancer types, providing a user‐friendly interface for accessing information about the global TME, for example, cell types, gene expression, and patient information [44]. More recently, another similar platform, ImmcanSCDB, was also developed for exploring the TME in a cancer‐type specific or universal manner [45]. The researchers collected data from 144 datasets across 56 cancer types and annotated the data with precise clinical, technological, and biological information, providing a freely accessible yet comprehensive platform. The development of such large‐scale databases has become more common as scRNA‐seq analyses of different cancer types become more mature; other examples include CancerSEA [46], Cancer Single‐cell Expression Map (CancerSCEM) [47], Characterising Tumour Subpopulations (CHARTS) [48] and CanCellVar [49]. All these studies aimed to integrate available datasets for the development of large‐scale platforms, allowing public access to examine the immune cell atlases in a more accessible way.

**TABLE 1:** Mainstream protocols used in scRNA‐seq studies.

Protocol	Single cell isolation and capture	Use of UMI	Reverse transcription	cDNA amplification	cDNA coverage	Ref.
Tang's method	Micromanipulation	No	Homopolymer tailing with terminal transferase	PCR	Nearly full length	[40]
CEL seq	Micromanipulation/limiting dilution	Yes	Use of a composite primer carrying a T7 promoter sequence	IVT for RNA, then PCR for cDNA	3′	[41]
SMART seq	FACS	No	Template switching by MMLV	PCR	Full length	[42]
SMART seq 2	FACS	No	Template switching by MMLV (improved with LNA‐containing TSO)	PCR (improved with KAPA DNA polymerase)	Full length	[43]
STRT seq	Micromanipulation (semi‐automated)/FACS	Yes	Template switching by MMLV	PCR	Full length	[36]
Drop seq	Microfludics (droplet‐based)	Yes	Template switching by MMLV	PCR	3′	[37]
CEL seq2	Microfludics (Fluidigm C1)	Yes	Use of a composite primer carrying a T7 promoter sequence	IVT for RNA, then PCR for cDNA	3′	[38]
10×	Microfludics (droplet‐based)	Yes	Template switching by MMLV	PCR	3′ or 5′	[39]

## 3 Discovery of Novel and Rare Immune Cell Types in the TME

A major implication behind the successful establishment of the comprehensive cell atlases of various types of cancer is the discovery of novel immune cell subsets or states. Based on the unique evolutionary trajectory of each cell in the TME, they form clusters with cells with similar origin in an unsupervised manner in scRNA‐seq [50], allowing researchers to identify rare populations via observation of the cell clusters, which was not possible with traditional analyses of bulk genomics [51]. Such discovery contrasts with the earlier view of the classic characterisation of basic immune cells; for example, effector T cell, naïve T cell, B cell, macrophage and dendritic cell. Many different novel immune cell types have been discovered as the subpopulations or rare cell states of these classic immune cell types with the aid of scRNA‐seq (Table 2). Therefore, a more comprehensive understanding of the composition and functions of the TME can be achieved.

**TABLE 2:** Summary of scRNA‐seq studies analysing the TME.

Cancer type	Tissue	Cell type	Protocols	Dataset availability	Key findings about the TME	Ref.
Breast cancer	Tumours and lymph node	All cells	Fluidigm C1 and Illumina HiSeq 2500	GSE75688	Identified three main groups of immune cells: B cells, T cells, and macrophages, mostly with immunosuppressive characteristics	[19]
Breast cancer	Tumours, normal tissue	CD45^+^ immune cells	InDrop and Illumina NextSeq	GSE114727, GSE114724, GSE114725	Revealed various immune cell subpopulations, such as T cell, B cell, and myeloid cell subpopulations Revealed T cell heterogeneity as a continuum of activation and differention states	[20]
LUAD, LUSC and NSCLC	Tumours and adjacent non‐malignant tissues	All cells	10× Genomics (V1 and V2) and Illumina Hiseq 4000	https://gbiomed.kuleuvenbe/scRNAseq‐NSCLC	Heavy enrichment of B cells was observed in the TME compared to other immune components Revealing different immune subpopulations and their features, such as low expression of CD8^+^ T cell marker genes linked with improved survival in LUAD, but worse survival in LUSC	[22]
Advanced NSCLC	Tumours	All cells	GEXSCOPE and Illumina HiSeq X10	GSE148071	Identification of some rare immune cell subtypes, such as follicular DCs and Th17 Greater abundance of exhausted T cells than cytotoxic T cells was observed	[22]
Melanoma	Tumours	All cells	Modified SMART‐Seq2 with Maxima Reverse Transcriptase	GSE72056	Revealed T cell exhaustion programs and their connection to T cell actiovation and clonal expansion	[52]
Melanoma	Tumours and PBMC	T cells and NK cells	MARS‐seq and Illumina Miseq	GSE123139	Showed a wide differentiation spectrum from early dysfuncational toward highly dysfunctional T cells	[53]
HNSCC	Tumours	All cells	SMART‐Seq2 and Illumina NextSeq	GSE103322	T cell exhaustion program was revealed Observed sigificant variations of CD8^+^ T cell proportions among patients	[54]
HCC	Tumours, adjacent liver tissues, hepatic lymph node, ascites, and PBMC	CD45^+^ immune cells	SMART‐Seq2 and Illumina Hiseq 4000	EGAS00001003449	Identification of two distinct macrophage states: THBS1^+^ macrohages and C1QA^+^ macrophages, enriched in tumours Observed LAMP3^+^ DCs to be the most active immune‐regulators of T cells and NK cells Revealed that LAMP3^+^ CDs could migrate from tumours to hepatic lymph nodes and induce T cell migration to tumours to exert effector function	[55]
PDAC	Tumours, normal contol pancreas from patients with other cancers and non‐malignant pancreatic tumour patients	All cells	10× Genomics V2 and Illumina HiSeq X10	CRA001160	Identification of different subtypes of T cells for example, CD8^+^ T cells with high expression of cytotoxic markers, CD8^+^ T cells with high expressioncell‐cycle‐related genes, CD4^+^ T cells with high expression of homing markers, CD4^+^ T cells with high expression of Treg signature genes Delineated macrophage diversity with various enriched features, such as ECM deposition and remodelling genes, MDSC migration chemokine gene, proinflammatory cytokines genes, and chemokine genes for T cell infiltration	[56]
Osteosarcoma	Tumours	All cells	10× Genomics (V2 and V3) and Illumina HiSeq X	GSE152048	High TIGIT expression was observed across different T cell subtypes and NK cells, suggesting potential use of TIGIT inhibition for treating osteosarcoma Observed three macrophage subsets: M1, M2, and M3 (FABP4^+^) alveolar macrophages from lung metastatic of osteosarcoma lesion	[57]
Pan‐cancer	1163 tumours across 24 cancer types	All cells	—	https://www.weizmann.ac.il/sites/3CA	The study focused on ITH of malignant cells, but still generated a large‐scale immune cell map, showing heavy infiltration of macrophages, T cells, and B cells	—
Pan‐cancer	1070 tumours across 30 cancer types	All cells	—	https://zenodo.org/records/10651059	Identification of gene signature and immune components of tertiary lymphoid structure Several cell states were observed to be associated with poor immunotherapy responses, for instance, CTSK^+^ macrophages	—
Pan‐cancer	Tumours from 716 patients across 24 cancer types	NK cells	—	GSE212890	Delineating NK cell heterogeneity in a cancer type‐specific manner, for example, enriched immature CD56^bright^CD16^lo^NK cells in NPC and basal cell carcinoma Identification of NK cell universal feature, such as reduced CD56dimCD16hi NK cell level in most cancer types	[26]
Pan‐cancer	Tumours from 210 patients across 15 cancer types	Myeloid cells	—	—	Revealing cancer‐specific characteristics of myeloid cells, such as greater abundance of TNF^+^ mast cells in NPC Showing shared myeloid cell features, such as prevalence of LAMP3^+^ cDCs in different cancers	[58]
Pan‐cancer	Tumours from 316 patients across 21 cancer types	T cells	—	GSE156728	Identification of various T cell subpopulations and functional states Identification of potential biomarkers or gene signatures associated with T cell subpopulations or functional states, for instance, FAT1 mutations are positively correlated with TNFRSFP^+^ Tregs	[28]
CRC	Tumours	All cells	10× Genomics V2, SMART‐seq2, and Illumina Hiseq 4000	GSE146771	Inferring prominent interactions between T cells and C1QC^+^ TAMs as well as 2 clusters of cDCs	[59]
CRC	Tumours	All cells	10× Genomics V2 and HiSeq 4000	GSE132465, GSE132257, and GSE144735	Inferring interactions between SPP1^+^ TAMs with CAFs and myofibroblasts via binding of SDC2 and MMP2	[60]
LUAD	Tumours	All cells	SureSelectXT Human All Exon V5 Illumina HiSeq 2500	GSE131907	The most significant interactions were observed between a novel cancer cell state, tS2, and monocyte‐derived macrophages	[61]
HNSCC	PBMC, tumours, and tonsil tissue	CD45^+^ immune cells	10× Genomics V2 and Illumina NextSeq 500	GSE139324	Inferring intercellular across different immune cells, including CD8^+^ T cells, DCs, B cells, NK cells, and mast cells	[62]
SCC	Tumours	All cells	10× Genomics V3 and HiSeq 4000	GSE144240	Inferring interactions between tumour‐specific keratinocytes with TAMs and MDSCs, such interactions contribute to tumour invasion	[63]
EAD	Tumours	All cells	10× Genomics and DESeq2	EGAS00001006469	Identified TMC as an indicator for immunotherapy outcome and prognosis	[64]
Ovarian cancer	Tumours	All cells	—	—	Observation of SPP1^+^ TAMs in chemotherapy‐resistant patients	[65]
Glioblastoma	Tumours	All cells	—	—	Revealing the superior effector function and reduced exhaustion responses of TLE4‐KO and IKZF2‐KO CAR T cells	[66]
ESCC	Tumours	All cells	10× Genomics V3 and Illumina HiSeq 3000	—	Observation of a range of infiltrating immune cells, such as T cells, NK cells, DCs and immunosuppressive TAMs after radiotherapy	[67]
Ovarian cancer	Tumours	All cells	10× Genomics V3 and Illumina NovaSeq 6000	GSE213243	Observation of enriched MDSC‐like myeloid population and suppressed γδ T cells after chemotherapy	[68]
PDAC	Tumours	All cells	10× Genomics (V2 and V3) and Illumina NovaSeq 6000	GSE205013	Reduction in inhibitory checkpoint molecule expression and interactions with CD8^+^ T cells	[69]

The roles and types of tumour infiltrating immune cells are very diverse, depending on the tissue types and the signals received [70]. Revealing rare immune cell types in the TME by scRNA‐seq can help dissect the complex TME and thus expand the options for therapeutic development. A case in point is the discovery of various macrophage subpopulations in the TME. The idea that TAMs exhibit M1 and M2 activation pathways, which are of anti‐cancer and cancer‐promoting properties respectively, is well accepted. While some macrophage subpopulations or states with potential therapeutic implications beyond the classical M1/M2 characterisation were further discovered by different researchers with the aid of scRNA‐seq [56]. In a recently published study, an exciting discovery of the macrophage‐myofibroblast transition (MMT) in TAMs has been reported; this began with the observation of an α‐SMA^+^ macrophage population in a scRNA‐seq dataset of NSCLC [71]. RNA velocity and pseudotime analysis of the scRNA‐seq dataset also revealed the development of CAFs from TAMs via MMT, providing valuable insight for further study in protumoral TAM or CAF. A more recent study from the same group discovered another rare macrophage subset, which is a neuron‐like macrophage subset developed via macrophage to neuron‐like cell transition (MNT) [72]. A Tubb3^+^ macrophage population was first observed in scRNA‐seq datasets of mouse TAM and NSCLC; subsequent in vitro and in vivo studies revealed the neuronal phenotype of this TAM subtype and its role in tumour innervation. Other groups also uncovered various TAM subpopulations; for instance, a SPP1^+^ macrophage subset was reported to be protumoral in CRC by scRNA‐seq [59, 73]. This SPP1^+^ TAM subset was shown to be distinct from the traditional classification of M1/M2 TAM and enriched with pro‐angiogenic signature genes. In another study, a group of CD73^hi^ macrophages was identified in glioblastoma by scRNA‐seq together with mass cytometry [74]. The CD73^hi^ macrophages were shown to exhibit high expression of immunosuppressive genes and inhibit T cell infiltration, leading to their findings of an effective combinatorial treatment of anti‐CD73 and dual blockade of PD‐1 and CTLA‐4. Apart from the studies mentioned, some pan‐cancer scRNA‐seq studies also provided valuable contributions in defining TAM diversity. Identification of various TAM subsets in these studies, with datasets of more than 10 cancer types, illustrated a comprehensive phenotypic map of TAM; for example, C1QC^+^ TAMs, LYVE1^+^ TAMs, TREM2 TAMs and IL4I1 TAMs, which were suggested to be correlated with cancer development via different mechanisms [27, 75]. Due to the increasing emergence of new TAM subsets being identified with the aid of scRNA‐seq, organisation of the data becomes crucial. Recently, Ma et al. reviewed more than 30 scRNA‐seq studies of TAM, TME and TME. They proposed that the TAM subsets identified in these studies can be classified into seven categories: interferon‐primed TAMs, immune regulatory TAMs, inflammatory cytokine‐enriched TAMs, lipid‐associated TAMs, pro‐angiogenic TAMs, resident‐tissue macrophage‐like TAMs and proliferating TAMs [76]. This information may provide insights for future study in TAM.

Tumour‐infiltrating T cell is another important player in the TME; different types of T cells have been widely reported with a significant association with cancer prognosis. In recent years, many studies have revealed the presence of different T cell subsets by scRNA‐seq; it has been observed that T cell composition and diversity vary across different cancer types [28]. For instance, a T cell subset, a group of CXCL13^+^BHLHE4^+^ T helper type 1 (Th1)‐like cells, was identified and observed to be enriched in microsatellite unstable tumour in a CRC dataset [77]. This CXCL13^+^BHLHE4^+^ Th1‐like subset was also noticed with elevated clonal expansion and proliferation. It was also suggested that there is a developmental connection between this subset and another T cell subset, GZMK^+^ effector memory T cells; therefore, it is possible that the CXCL13^+^BHLHE4^+^ Th1‐like subset is a particular T cell developmental state. In another study, a new population of T cell was also discovered by scRNA‐seq; it is known as αβ T cell receptor (TCR)‐positive FCER1G‐expressing innate‐like T cell with high cytotoxic potential (ILTCKs) [78]. This subset was characterised by high expression of Gzmb, Fcer1g and Klrb1c, which is a gene that encodes for NK1.1, the phenotypic marker of NK cells. In contrast with conventional cytotoxic CD8^+^ T cells, ILTCKs were observed to be developed from a different progenitor and exhibited a stronger anti‐tumour effect. Such discovery was suggested to be a potential therapeutic option. In a pan‐cancer analysis of tumour‐infiltrating T cells, a few T cell subsets were revealed by scRNA‐seq [79]. The tumour‐infiltrating T cells used in this study were obtained from more than 300 patients; some newly discovered T cell subsets, for example, KIR^+^ NK‐like T cells, ZNF683^+^CXCR6^+^ tissue‐resident memory T cells and GZMK^+^ effector memory T cells [28]. Further analysis of T cell development with RNA velocity was performed; these T cell subsets were suggested to be transitional states to T cell exhaustion, which is a state of hypofunction with the loss of T cell effector function [80]; in other words, it is a state with loss of anti‐cancer ability. As scRNA‐seq has promoted the discoveries of novel tumour‐infiltrating T cell subset or state, a more comprehensive and systematic characterisation of these newly discovered T cells becomes pivotal for future research in tumour‐infiltrating T cells in addition to the conventional T cell characterisation. This is exemplified in the work of Van der Leun et al., in which these newly discovered T cells from nine scRNA‐seq studies of tumour‐infiltrating T cells were summarised with information regarding functional characteristic, gene signature, clonality and so forth [81].

Apart from macrophages and T cells, novel subtypes or cell states of other cancer‐associated immune cells have also been identified by scRNA‐seq in recent years. One example is the discovery of CLEC9A^+^ DCs in NPC [82]; the CLEC9A^+^ DCs are reported to be negatively correlated with the DNA level of Epstein–Barr virus (EBV), which has been suggested to be a contributing factor to the development of nasopharyngeal carcinoma [83]. While details of the relationship between CLEC9A^+^ DCs and EBV‐related NPC are yet to be elucidated, in another study, a novel DC subset was also discovered in patients with HCC. Using scRNA‐seq, a cluster of LAMP3^+^ DCs was identified; this DC subset did not fall into any classic DC characterisation and was reported to have the highest expression of ligands that interact with receptors of T cells and NK cells for regulatory functions [55]. In a NSCLC study, the authors aim to reveal the diversity of B cells with scRNA‐seq and successfully identified a few novel B cell subtypes, for example, CD20‐CD19‐CD79A^+^CD79B^+^ B cells and BCMA^+^ IgG^hi^ B plasma cells [84]. The roles of some of these B cell subtypes in NSCLC were studied, such as the ability to promote cell proliferation in late stages of NSCLC by the BCMA^+^ IgG^hi^ B plasma cells.

**FIGURE 3:**
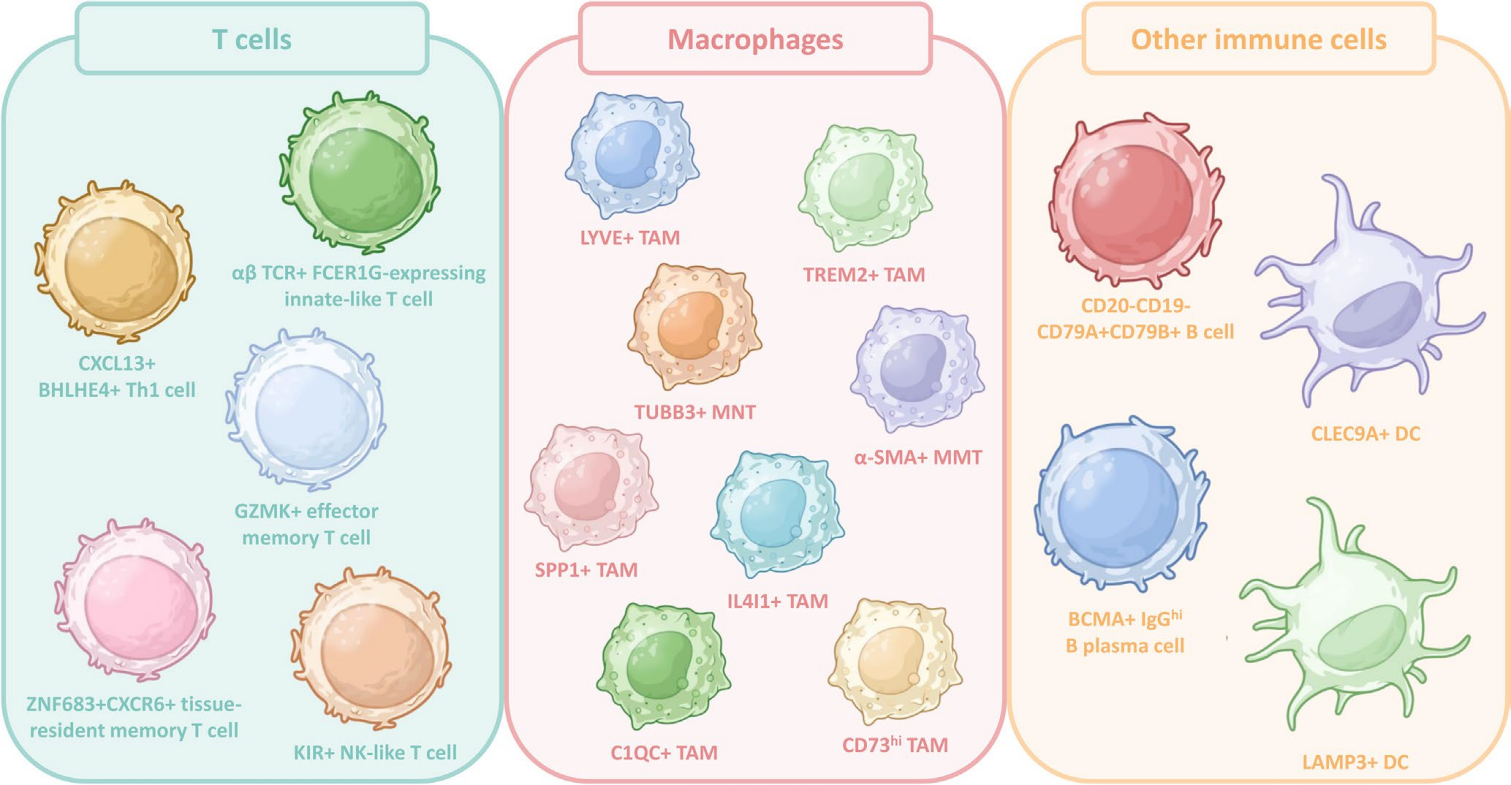
Identification of rare and novel immune cell subtypes in the TME by scRNA‐seq. Different rare and novel immune cell types in the TME were identified by scRNA‐seq, including TUBB3^+^ MNT, α‐SMA^+^ MMT, SPP1^+^ TAM, CD73^hi^ TAM, C1QC^+^ TAM, TREM2^+^ TAM, LYVE^+^ TAM, IL4I1^+^ TAM, +FCER1G‐expressing innate‐like T cell, CXCL13^+^BHLHE4^+^ Th1 cell, ZNF683^+^CXCR6^+^ tissue‐resident memory T cell, KIR^+^ NK‐like T cell, GZMK^+^ effector memory T cell, CD20‐CD19‐CD79A^+^CD79B^+^ B cell, BCMA^+^ IgGhi B plasma cell, CLEC9A^+^ DC, and LAMP3^+^ DC.

These findings demonstrate that scRNA‐seq has enabled the identification of a wide spectrum of novel immune cell subtypes in the TME (Figure 3), contributing to a more nuanced understanding of the immune landscape in cancer and revealing potential targets for future therapeutic development.

## 4 scRNA‐Seq Reveals Temporal Dynamics of the TME

The dynamic and heterogeneous nature of the TME is largely attributed to the highly plastic and constantly evolving tumour‐infiltrating immune cells [85, 86]. It is now widely recognised that the majority of these immune cells are in transient states instead of discrete developed states [73]. Dissection of the cell fate of tumour‐infiltrating immune cells offers a deeper and more comprehensive insight into the TME; this can be accomplished using a range of scRNA‐seq analysis tools, such as Monocle [87, 88], PAGA [89], Slingshot [90], RNA velocity [91, 92] and so forth. These tools aim to elucidate the pseudotime analysis as well as trajectory inference of cells, each demonstrating varied performance and distinct underlying principles [93].

It has been shown by many studies that macrophages are a highly plastic and versatile population in the TME by scRNA‐seq; the analysis tools for pseudotime and trajectory inference have been widely used to study the dynamics of macrophages in the TME. A case in point is a scRNA‐seq study of macrophages in lung adenocarcinoma, where the authors discovered the origin of alveolar and interstitial macrophages. According to pseudotime analysis by Monocle, the majority of interstitial macrophages were found to have originated from blood monocytes, whereas alveolar macrophages were mainly self‐renewed [94]. They also observed the development of M1‐like to M2‐like phenotypes in TAMs with pseudotime. In another study, TAMs in liver metastatic tumours were investigated according to their metabolic patterns [95]. As mentioned previously, discoveries of novel TAM subsets, which undergo MMT and MNT, are examples showing the heterogeneity of TAM. The research group utilised Monocle and RNA velocity to illustrate that the novel myofibroblast‐like and neuron‐like macrophages are derived from macrophages under the stimulation of specific factors secreted by the TME [71, 72].

Another highly abundant and heterogeneous immune cell type in the TME is T cell; many studies have shown that exhausted T cells are common in the TME and important for cancer progression. By employing scRNA‐seq and pseudotime analysis, Guo et al. illustrated the developmental pathways of T cells in NSCLC. They showed that naïve T cells branched into two distinct trajectories of CD8^+^ T cells, represented by Monocle components of T cell exhaustion and gain of cytotoxicity [96]. The developmental trajectory of CD4^+^ T cells was also analysed, showing branched developments toward effector T cells and exhausted T cells with T follicular helper‐like features. In another scRNA‐seq study of lung adenocarcinoma, developmental trajectories of CD8^+^ T cells were shown to be similar to the study by Guo et al. Three distinct branches representing different states: naïve, cytotoxic and exhausted were observed, with each Monocle component correlating with cytotoxicity and exhaustion [61]. The observation of the enrichment of exhausted T cells in the late period of pseudotime is not only observed in these lung cancer studies, but also in other cancers by pseudotime analysis [97, 98].

The pseudotime analysis tools have also been widely used to infer the developmental pathways of other TME components. For instance, RNA velocity and Monocle analysis were used to illustrate the role of Smad3 in polarisation of N1/N2 states of neutrophils, indicated by enriched Smad3 expression in the N2 developmental pathway; N1 development was observed in Smad3‐knockout (KO) neutrophils [99]. Another example is a study of myeloid‐derived suppressor cells (MDSCs) in breast cancer; MDSCs are a group of alternatively activated immature immune cells with strong immunosuppressive activity. It has been suggested that elucidation of their developmental mechanisms can be useful for developing therapeutic strategies that reverse their immunosuppressive activities [100]. scRNA‐seq of MDSCs isolated from spleens of breast cancer‐bearing mice revealed distinct clusters of MDSCs and their normal counterparts, neutrophils and monocytes [101]. Pseudotime analysis was employed to further elucidate the development of granulocytic‐MDSCs (G‐MDSCs); it was shown that G‐MDSCs and neutrophils emerge through different developmental pathways from neutrophil progenitors, represented by a three‐branch trajectory generated by Monocle.

## 5 scRNA‐Seq Reveals Intercellular Interactions Involving the TME

Along the process of cancer progression from pre‐malignant state to malignant state, or even metastasis, cell–cell interactions are essential elements for such progression [102]. Such interactions are so diverse that they include the involvement of pre‐malignant cells, malignant cells, as well as non‐malignant cells, including but not limited to immune cells. A high degree of heterogeneity of the TME induces the formation of a highly complex interaction network; this is driven by a variety of cellular interactions such as ligand–receptor interaction and paracrine signalling [103]. Many events that take place during cancer progression rely on these intercellular communications; therefore, a deeper understanding of this complex interaction network may provide new insights into the development of new therapeutic opportunities [104]. Analysis of such cellular interactions has been aided by the advent of scRNA‐seq due to the development of analysis methods for the inference of intercellular interactions. Some well‐known examples of these tools include CellPhone DB [105], CellChat [106] and NicheNet [107], which are all commonly used in recent studies. There are still many other similar tools available for research use; in general, all these tools are comprised of two main components: a database with prior knowledge of ligand–receptor interactions and a prediction method to estimate intercellular interactions based on the ligand or receptor expression obtained from scRNA‐seq data [108, 109].

The cellular interaction analysis tools have already been used to dissect intercellular interactions in many different studies; many of these interactions involve the participation of tumour‐associated immune cells. To exemplify, a scRNA‐seq study identified that C1QC^+^ TAMs and 2 clusters of cDCs interacted more prominently with T cells in CRC patients via CellPhone DB [59]. Apart from this, the newly discovered SPP1^+^ TAMs, as mentioned previously, were observed to be interacting with CAFs and myofibroblasts via the binding of SDC2 and MMP2. Interactions of SPP1^+^ TAMs were also examined in another study of CRC, where the SPP1^+^ TAMs were found to exhibit interactions with tumour cells via the binding of SPP1 and CD44 on tumour cells [60].

In the study where LAMP3^+^ DCs were identified as mentioned in the previous section, intercellular interactions of LAMP3^+^ DCs were also identified in HCC. The greatest number of ligands and receptors was observed in DCs and T cells respectively among myeloid and lymphoid cells [55]. In particular, the greatest expression of ligands was observed in LAMP3^+^ DCs among all DCs, and the effects of their interactions appeared to be relatively diverse. There seemed to be a dual role of NK cell regulation in LAMP3^+^ DCs, in which the expression of NECTIN2 can interact with CD226 on circulating NK cells or TIGIT on liver resident NK cells for activating signal and inhibiting signal respectively. Apart from NK cells, DCs were shown to be interacting with T cells via a range of migration chemokines; expressions of PD‐L1 and PD‐L2 were also observed; they were predicted to be interacting with various types of T cells via binding to PD‐1.

In a LUAD study, a novel cancer cell state, tS2, was identified, and the most significant interactions in the early stage of lung tumour were observed between tS2 and monocyte‐derived macrophages; it was suggested that these interactions were involved with growth factors signalling, for example, VEGFA and VEGFB [61]. While the most prominent interactions were observed between monocyte‐derived macrophages and exhausted CD8^+^ T cells, the signals delivered to the exhausted CD8^+^ T cells included both activating and inhibitory signals. In another lung cancer study where samples from advanced NSCLC patients were used, intercellular interactions involving various types of immune cells were identified. For example, cancer cells with expressions of CXCL1, CXCL2, CXCL3 and CXCL8 were suggested to be interacting with neutrophils via binding to CXCR1 and CXCR2 [22]. Increased interactions between DCs and T cells were also observed in LUAD and LUSC patients; CXCR3 and its ligands were also involved in the interactions, which may suggest strong effector T cell recruiting and trafficking. Immunosuppression was also revealed by the interactions between TAMs and T cells, in which T cell functions were inhibited via checkpoint pathways.

For the studies discussed above, CellPhone DB was used for inferring intercellular interactions involved in the TME, while as mentioned earlier, there are other intercellular interaction analysis tools available and have been used to study the TME. One example is the use of NicheNet to study the leading edges of the fibrovascular niche in human squamous cell carcinoma, in which tumour‐specific keratinocyte receptors were found to be interacting with ligands expressed on TAMs and MDSCs; such cellular crosstalk is associated with epithelial‐mesenchymal transition and epithelial tumour invasion [110]. Another example is the development of CellTalker by Cillo et al., who also used this tool to study the TME of HNSCC [62]. Intercellular interactions across a range of tumour‐associated immune cells, such as CD8^+^ T cells, DCs, B cells, NK cells and mast cells, were detected. The pattern of interactions between immune cells was observed to be different between human papillomavirus (HPV)+ and HPV− HNSCC, indicating unique interactions participated in a specific type of HNSCC.

## 6 Clinical Implications of scRNA‐Seq Data Regarding TME

Increasing evidence has shown that the immune system is compromised to facilitate cancer development, a phenomenon known as ‘evading immune destruction’, which is considered one of the cancer hallmarks [4]. Immune evasion is driven by the immunosuppressive nature of the TME, which includes a variety of dysfunctional and immunosuppressive immune cells, such as TAMs, MDSCs and exhausted T cells [111]. A deeper understanding of the mechanisms underlying this immunosuppressive environment has led to the revolutionary development of immunotherapies. These therapies aim to restore the natural defence of the immune system for elimination of malignant cells, a prime example is the anti‐PD‐1/PD‐L1 therapies, which have become some of the most widely used cancer treatments [112]. However, there are still limitations to immunotherapies, such as low response rates in several common cancer types and resistance toward immunotherapies [113, 114]. Traditional therapies like chemotherapy and radiotherapy also face similar challenges [115]. As discussed in previous sections, scRNA‐seq has provided unprecedented insights into the complexity and dynamics of the TME. These insights hold significant clinical implications for improving patient stratification and predicting treatment responses for current cancer therapies [114, 116]. Efforts to identify novel therapeutic targets and develop personalised treatment strategies are also major focuses of cancer therapy development, with scRNA‐seq contributing to these applications.

Predicting responses to immunotherapies during the treatment decision process remains a significant challenge in clinical practice [117]. Traditional models of patient stratification, for example, screening for high expression of immune checkpoints in patients for immune checkpoint inhibitor (ICI) therapies [118, 119], have shown limitations [120]. With the use of scRNA‐seq, many studies have identified potential biomarkers to predict responses to currently available immunotherapies. To exemplify, a team used results obtained from scRNA‐seq to study the correlation of cell‐type frequencies across NSCLC tumours and discovered a group of cells with high correlations, which are T‐activated cells, IgG^+^ plasma cells and certain monocyte populations, collectively known as lung cancer activation module (LCAM) [121]. It was then observed that a high LCAM score was associated with an enhanced response to immunotherapy. In a melanoma study, it was suggested that the transcription factor TCF7 in CD8^+^ T cells was associated with improved response to ICI [97]. scRNA‐seq was performed on 48 melanoma samples from patients treated with ICI; two major cell states of CD8^+^ T cells were identified, one of which is a TCF7^+^ group that showed upregulation of genes related to memory, activation and survival [97]. They further validated that the presence of TCF^+^CD8^+^ T cells in both baseline and post‐treatment was associated with enhanced ICI response through immunohistochemistry staining. A recent study based on a phase I/II clinical trial also demonstrated the use of scRNA‐seq to identify biomarkers for immunotherapy response [64]. Thirty‐eight oesophageal adenocarcinoma patients were treated with ICI followed by combinatory treatment of ICI and chemotherapy; biopsies collected at the end of the treatment protocol were used for scRNA‐seq. From the scRNA‐seq results, a cluster with high tumour monocyte content (TMC) was identified, which was associated with improved clinical benefits and overall survival. Further analyses showed that high TMC was linked to better clinical outcomes. TMC was also suggested to be used as a complementary biomarker with tumour mutational burden, a common predictor for immunotherapy response, to provide a more accurate prediction of clinical response to ICI and chemotherapy combinatorial treatment [64].

In addition to immunotherapy, scRNA‐seq findings that are related to the TME also help to predict responses to conventional therapies. For instance, a scRNA‐seq study emphasised the importance of non‐tumour cell components, especially the TME, in directly influencing chemotherapy responsiveness in ovarian cancer patients. They observed enriched SPP1^+^ TAMs in chemotherapy‐resistant patients, suggesting SPP1^+^ TAMs directly interact with CD44 on tumour cells to promote chemoresistance [65]. While the identification of another cell type, GZMA^hi^ lymphocytes, was found in chemotherapy‐sensitive patients with better prognosis. As for radiotherapy, Jang et al. applied scRNA‐seq to study the TME in breast cancer and its relationship with radiosensitivity [122]. They found that radiotherapy resistant (RR) tumour cells not only carry a high tumour mutation burden with a microsatellite instability signature, but also express elevated levels of PD‐L1. This upregulation of PD‐L1 coincides with an increased number of PD‐L1, PD‐1 and CTLA 4‐CD80/86 ligand‐receptor pairs between tumour cells and infiltrating T cells, particularly in the TNBC and HER2 subtypes, suggesting that checkpoint mediated crosstalk dampens anti‐tumour T cell activity and promotes radiotherapy resistance. By contrast, radiotherapy sensitive (RS) cells exhibit lower PD‐L1 expression, fewer inhibitory checkpoint interactions, and a predominance of mismatch repair–related mutations. These findings highlight PD‐L1 upregulation and enhanced interactions between cancer cells and T cells as immune microenvironment indicators of poor radiotherapy response, and they provide a rationale for combining PD‐1/PD‐L1 blockade with radiotherapy to overcome radiotherapy resistance.

scRNA‐seq has been used to identify new targets within the TME, offering potential therapeutic benefits and insights to improve current immunotherapies. As mentioned previously, a group of CD73^hi^ macrophages was identified in glioblastoma by scRNA‐seq; such discovery led to the discovery of an effective combinatorial treatment of anti‐CD73 and dual blockade of PD‐1 and CTLA‐4 [74]. Another glioblastoma study utilised scRNA‐seq to examine the effects of genetically edited chimeric antigen receptor (CAR) T cells, aiming to boost the anti‐cancer activity. Initially, a whole‐genome clustered regularly interspaced short palindromic repeats (CRISPR) KO screen identified genes linked to resistance in CAR T effector functions, with TLE4 and IKZF2 observed to be the most significant genes according to further in vitro studies [66]. Subsequent scRNA‐seq of TLE4‐KO and IKZF2‐KO CAR T cells revealed that the transcriptional profiles of these genetically modified CAR T cells exhibited superior effector function and reduced exhaustion responses, suggesting a promising approach for cancer treatment. In a study of melanoma CD8^+^ T cells, several subpopulations of CD8^+^ T cells were identified by scRNA‐seq, each with distinct characteristics; for example, cytotoxic subpopulation 3 was associated with better clinical outcomes, while exhausted subpopulation 2 was linked to poor prognosis [123]. The study also indicated that the TME when exhausted subpopulation 2 was the most abundant could be the optimal time for ICI treatments due to high expression of immune checkpoints. Additionally, three overexpressed genes (PMEL, TYRP1 and EDNRB) were identified in the exhausted subpopulation 2; these genes were observed to be associated with poor prognosis and proposed as potential targets for melanoma therapy.

scRNA‐seq has also been widely used to explore the use of combinatorial treatments of current immunotherapies or conventional cancer therapies with current immunotherapies at the transcriptomic level. A recent study employed scRNA‐seq to analyse transcriptomic changes in NSCLC tumour samples from 15 patients (*n* = 5) after receiving combinatorial treatment of PD‐1 blockade and chemotherapy [124]. The group observed expansion and activation of cytotoxic T cells, NK cells, and effector T cell phenotypes in memory CD8^+^ T cells, along with reduced immunosuppressive Tregs in patients who received treatments. Changes in macrophage composition were also observed, with increased tissue‐resident macrophages and anti‐tumour TAMs following combinatory treatment. While differences between MPR and NMPR groups among treated patients were also observed, including a reduction in an aged CCL3^+^ neutrophil subset in MPR patients, these neutrophils were predicted to interact with SPP1^+^ TAMs to induce poor therapy response. In another study of PDAC, scRNA‐seq was applied to examine tumour lesions in patients with and without prior chemotherapy treatment [69]. Changes in the PDAC TME were observed, primarily a reduction in inhibitory checkpoint molecule expression and interactions with CD8^+^ T cells, potentially leading to further resistance to immunotherapy.

Further scRNA seq studies have also been done to examine the influences of conventional therapies in remodelling the TME. In oesophageal squamous cell carcinoma (ESCC), radiotherapy was shown to induce the infiltration of diverse immune cells, including T cells, NK cells and dendritic cells, but most notably led to an accumulation of macrophages expressing immunosuppressive genes such as PD‐L1, SIRPA and IDO1, suggesting the emergence of a suppressive TME post‐treatment [67]. Similarly, in breast cancer, radiotherapy altered both tumour and immune compartments; while some effector T cell subsets were depleted, others such as naive‐like CD4^+^ T cells and early activated CD8^+^ T cells were expanded, alongside increased infiltration of myeloid cells, highlighting the dynamic nature of radiotherapy‐induced immune remodelling [125]. Chemotherapy‐induced changes were also evident in LUAD, where scRNA‐seq revealed a shift in macrophage phenotypes toward a metabolically reprogrammed, pro‐tumour ARG1^+^ state, along with enhanced activation of cytotoxic T and B cells [126]. In relapsed ovarian cancer, chemotherapy remodelled both the local and systemic immune compartments: the ascitic fluid was enriched in MDSC‐like myeloid populations and metabolically suppressed γδ T cells, while the peripheral blood showed signs of CD8^+^ T cell exhaustion, yet retained dominant TCR clones and diversified B cell receptor repertoires [68]. In a study of PDAC, scRNA‐seq was applied to examine tumour lesions in patients with and without prior chemotherapy treatment [69]. Changes in the PDAC TME were observed, primarily a reduction in inhibitory checkpoint molecule expression and interactions with CD8^+^ T cells, potentially leading to further resistance to immunotherapy. Collectively, these scRNA‐seq findings underscore how conventional therapies, beyond targeting cancer cells, can dramatically reshape the TME in ways that influence subsequent immunotherapeutic outcomes.

**FIGURE 4:**
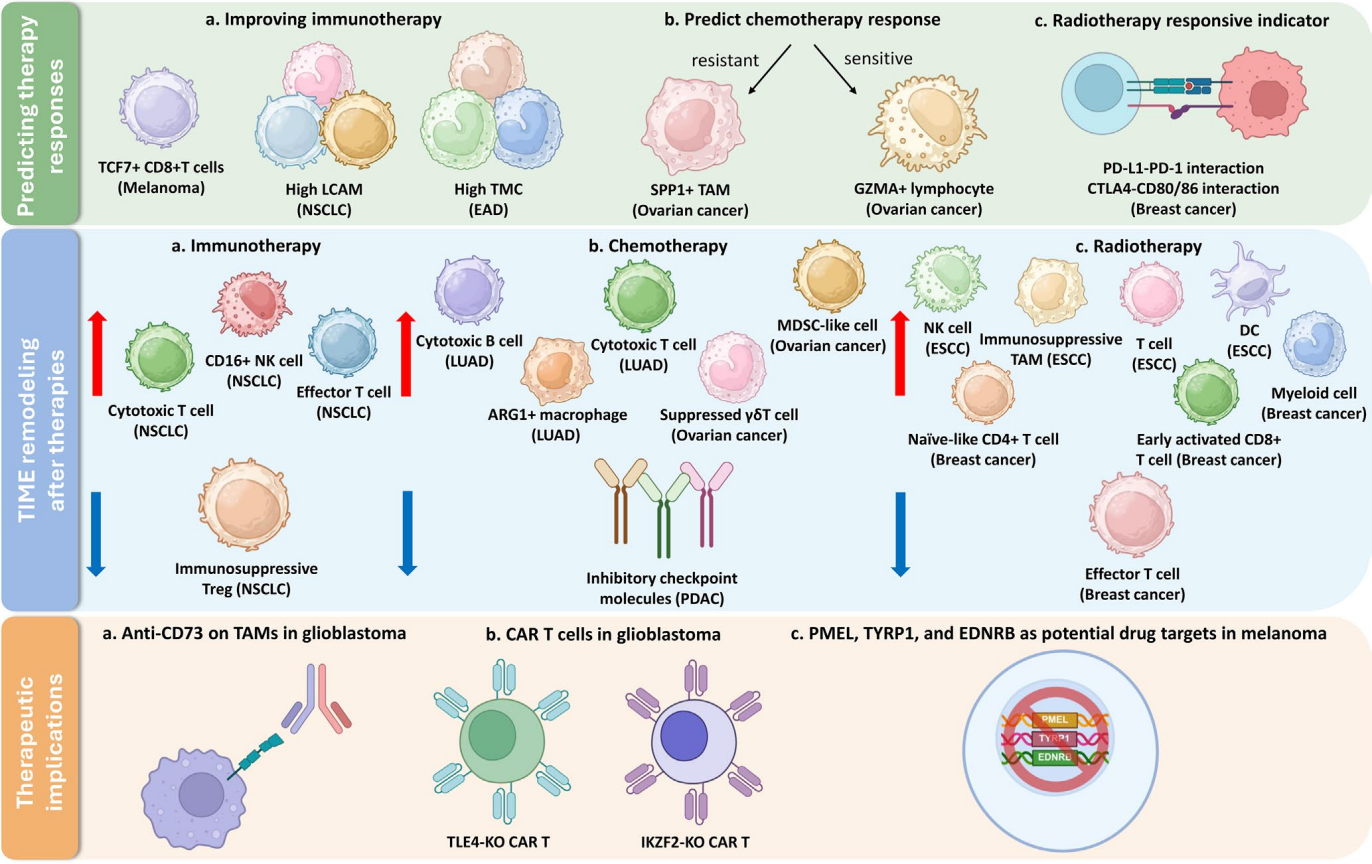
Clinical implications of TME discoveries by scRNA‐seq. This figure summarises key TME‐related findings by scRNA‐seq, highlighting their clinical implications in 1. Predicting therapy response: (a) Enrichment of TCF7^+^ CD8^+^ T cells in melanoma, high LCAM scores in NSCLC, and high TMC in EAD are associated with improved immunotherapy outcomes. (b) Chemotherapy response can be predicted by the presence of SPP1^+^ TAMs (resistance) or GZMA^+^ lymphocytes (sensitive) in ovarian cancer. (c) In breast cancer, the upregulation of immune checkpoints is linked to poor radiotherapy response. 2. TME remodelling after therapies: (a) Immunotherapy induces expansion of cytotoxic and effector cells, while reducing immunosuppressive Tregs in NSCLC. (b) Chemotherapy alters immune composition across cancers, increasing cytotoxic and MDSC‐like cells, ARG1^+^ macrophages and suppressing γδ T cells. (c) Radiotherapy leads to dynamic immune reshaping in ESCC and breast cancer, including accumulation of immunosuppressive TAMs, DCs and activated T cells. 3. Therapeutic implications: (a) Targeting CD73^+^ TAMs in glioblastoma. (b) CRISPR‐based KO of TLE4 or IKZF2 enhances CAR T cell function in glioblastoma. (c) PMEL, TYRP1 and EDNRB are highly expressed in exhausted CD8^+^ T cell subpopulations in melanoma and represent potential therapeutic targets.

These studies underscore the critical role of scRNA‐seq in advancing our understanding of the TME and improving cancer treatment strategies (Figure 4). By identifying key cellular and molecular landscapes, scRNA‐seq provides valuable insights that can inform the development of more effective and personalised therapies.

## 7 Conclusion and Future Prospects

scRNA‐seq has revolutionised our understanding of the TME by generating comprehensive immune cell atlases across different cancer types. These atlases have unveiled the intricate landscape of the TME, offering unprecedented insights into cellular heterogeneity, novel or rare cell subsets, temporal dynamics and intercellular interactions. The information obtained from these scRNA‐seq studies holds significant clinical implications, particularly in biomarker identification, patient stratification, and the discovery of novel therapeutic targets. Consequently, scRNA‐seq presents opportunities for cancer precision medicine, addressing the challenges of current cancer treatments due to the heterogeneous and unique nature of the TME across patients.

Despite its significant contributions to elucidation of the TME that leads to improvements in cancer treatment strategies, there are still limitations that can be addressed with the integration of other advanced technologies. An emerging technique, spatial transcriptomics, has gained popularity in recent years and is expected to become more commonly used; for example, GeoMx developed by nanoString and Visium developed by 10× Genomics [127]. Spatial transcriptomics analyses the spatial organisation of the TME, overcoming the loss of spatial context due to tissue dissociation for scRNA‐seq [128]. Although scRNA‐seq also provides very limited spatial information by inferring intercellular interactions, the integration of scRNA‐seq with spatial transcriptomics can provide a more comprehensive understanding of the native spatial architecture within the TME. Multi‐omics integration represents another frontier in enhancing the utility of scRNA‐seq data. Combining scRNA‐seq with other omics technologies that profile genome, epigenome, proteome, metabolome and other omics provides a more holistic understanding of cellular states and functions within the TME [129]. For instance, integrating scRNA‐seq with assay for transposase‐accessible chromatin sequencing (ATAC‐seq) can elucidate relationships between chromatin accessibility and gene expression, providing insights into gene regulatory mechanisms [130]. The integration of scRNA‐seq with cellular indexing of transcriptomes and epitopes by sequencing (CITE‐seq) can allow simultaneous multiplexed protein‐marker detection and transcriptome profiling [131] and lead to more refined cell type and state classifications, potentially revealing functional differences that are not apparent from transcriptomics alone.

In the future, the continuous development of bioinformatics tools is anticipated to provide more sophisticated tools and tools that infer some new cellular features which could not be achieved in the past [132]. The applications of artificial intelligence and machine learning (AI/ML) algorithms to scRNA‐seq data analysis are expected to offer the potential for more efficient and accurate processing of the vast amounts of data [133, 134]. For example, machine learning algorithms can be trained to classify cell types based on their gene expression profiles with higher accuracy, potentially outperforming traditional clustering methods [135]. These advanced computational approaches may also improve biomarker identification and drug response prediction, ultimately contributing to improved patient stratification and personalised treatment strategies [133]. Another trend to be looking forward to is the integration of scRNA‐seq into clinical practice for patient stratification, treatment selection and therapy response monitoring [136]. As scRNA‐seq technologies become more cost‐effective and streamlined, this expectation is becoming increasingly feasible. The transition from research tool to clinical application represents a significant step towards realising the full potential of scRNA‐seq in oncology.

In conclusion, while scRNA‐seq has already significantly deepened our understanding of the TME and improved cancer treatment strategies, its integration with spatial transcriptomics, multi‐omics approaches and AI/ML promises to further revolutionise our approach to cancer research and treatment. As these technologies continue to evolve, they hold the potential to significantly enhance our ability to diagnose, treat, and monitor cancer, ultimately improving patient outcomes and having the potential to enable truly personalised treatment strategies tailored to each patient's unique tumour immune landscape.

## Author Contributions

**Jane Siu‐fan Li:** writing – original draft (equal), writing – review and editing (equal). **Zoey Zeyuan Ji:** writing – original draft (equal), writing – review and editing (equal). **Aaron Qi Zhang:** writing – review and editing (supporting). **Calvin Sze‐Hang Ng:** writing – review and editing (supporting). **Guibin Qiao:** writing – review and editing (supporting). **Dongmei Zhang:** writing – review and editing (supporting). **Chunjie Li:** writing – review and editing (supporting). **Qing Zhang:** writing – review and editing (supporting). **Ka‐Fai To:** writing – review and editing (supporting). **Patrick Ming‐Kuen Tang:** supervision (lead), writing – original draft (lead), writing – review and editing (lead).

## Conflicts of Interest

The authors declare no conflicts of interest.

## Data Availability

No new data were generated or analyzed in this study. All data discussed are derived from previously published sources, which are appropriately cited within the manuscript.
